# Remodeling tumor microenvironment with natural products to overcome drug resistance

**DOI:** 10.3389/fimmu.2022.1051998

**Published:** 2022-11-10

**Authors:** Wanlu Zhang, Shubo Li, Chunting Li, Tianye Li, Yongye Huang

**Affiliations:** ^1^ College of Life and Health Sciences, Northeastern University, Shenyang, China; ^2^ Liaoning Center for Animal Disease Control and Prevention, Liaoning Agricultural Development Service Center, Shenyang, China

**Keywords:** tumor microenvironment, natural products, drug resistance, cancer stem cells, p53, traditional Chinese medicine

## Abstract

With cancer incidence rates continuing to increase and occurrence of resistance in drug treatment, there is a pressing demand to find safer and more effective anticancer strategy for cancer patients. Natural products, have the advantage of low toxicity and multiple action targets, are always used in the treatment of cancer prevention in early stage and cancer supplement in late stage. Tumor microenvironment is necessary for cancer cells to survive and progression, and immune activation is a vital means for the tumor microenvironment to eliminate cancer cells. A number of studies have found that various natural products could target and regulate immune cells such as T cells, macrophages, mast cells as well as inflammatory cytokines in the tumor microenvironment. Natural products tuning the tumor microenvironment *via* various mechanisms to activate the immune response have immeasurable potential for cancer immunotherapy. In this review, it highlights the research findings related to natural products regulating immune responses against cancer, especially reveals the possibility of utilizing natural products to remodel the tumor microenvironment to overcome drug resistance.

## 1 Introduction

**Figure 1:**
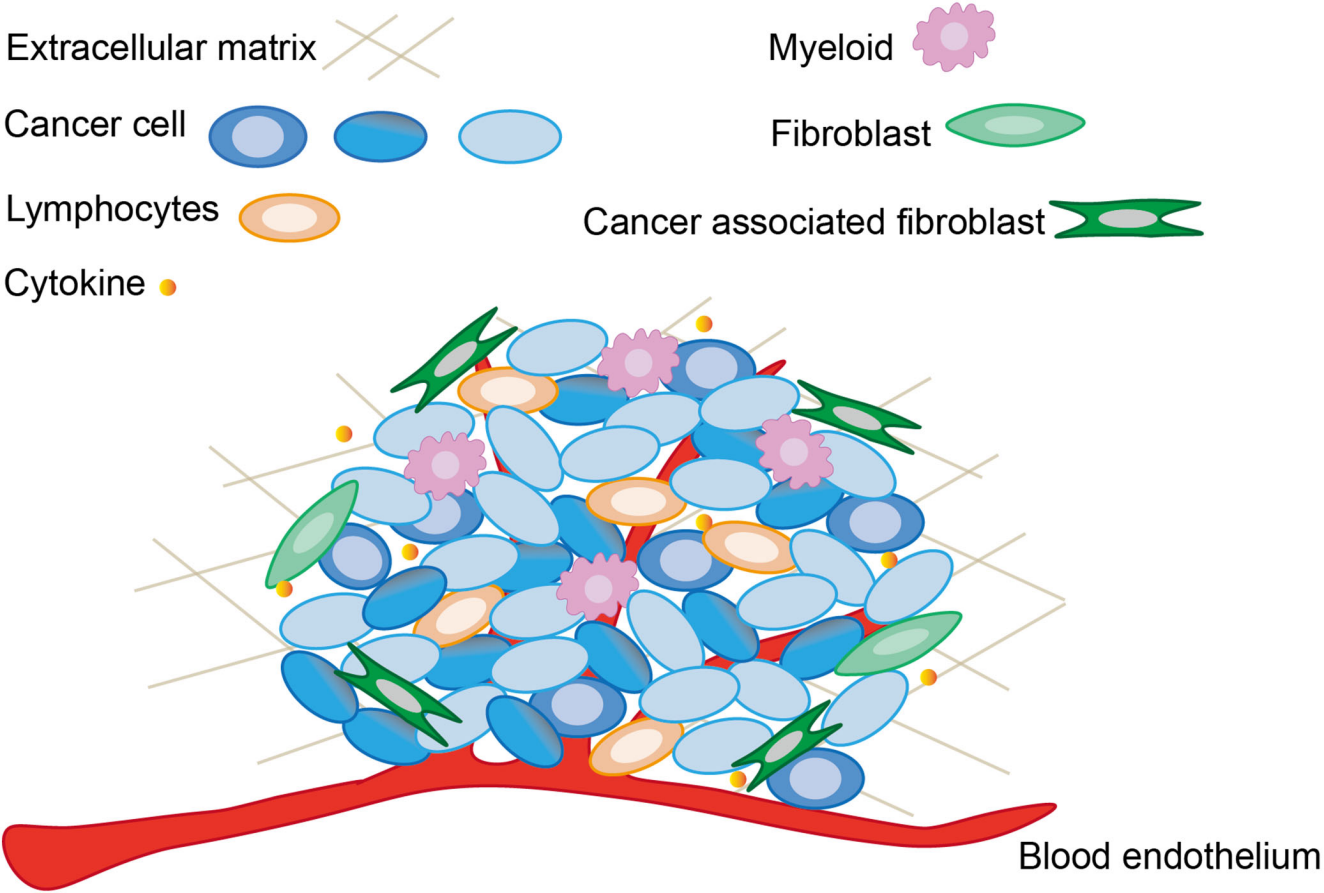
The main components and associated cells within tumor microenvironment. The tumor microenvironment not only contains cancer cells and already altered cellular structures, but is also capable to recruit normal cells and release cytokines to sustain the function of tumor microenvironment.

The incidence of cancer has been rising worldwide and is becoming a major threat to human life and health. According to a recent report on global cancer statistics, cancer is the third or fourth leading cause of death in 23 countries and the first or second leading cause of death by age 70 in 112 of 183 countries (1). The hallmarks of cancer have been proposed as a set of functions acquired by normal cells transitioning to neoplastic growth states that are essential capabilities for their ability to form malignant tumors (2). Cancer cells could acquire the following hallmarks by recruiting normal cells to create a tumor microenvironment: evade growth suppressors, resist cell death, sustain proliferative signals, induce angiogenesis, enable replication immortal, activate invasion and metastasis, deregulate cellular energetics, avoid immune destruction, acquire tumor-promoting inflammation, and generate genome instability and mutation (3). In 2022, four new hallmarks of cancer have been proposed: epigenetic changes that can affect gene expression, the ability of cells to regress from a specific specialized functional state, the role of microorganisms, and neuronal signal (2). The origin of cancer is extremely complex. The homeostasis system within body prevents excessive cell proliferation, and the development of normal cells into malignant tumors depends on a variety of inhibitory mechanisms (4). With deeper research into the mechanisms of cancer and more fully understanding mechanisms of cancer development and malignant transformation, the hallmarks of cancer are increasingly becoming logical science as an integrative concept (2). In another word, the original progression underlying tumorigenesis and further fate of cancer development might be all in the control of “tumor microenvironment” (**Figure 1**).

At the present time, the clinical treatments used for cancer commonly include surgical resection, radiation therapy, chemotherapy, targeted molecular therapy, immunotherapy, and traditional Chinese medicine. Among these, cancer immunotherapy coordinates the body’s immune system to target and obliterate cancer cells by producing a lasting anti-tumor response, which can more reduce cancer cell metastasis and cancer recurrence than traditional treatment methods (5). Tumor microenvironment targets the local immune response in solid tumors to help immune cells entering the tumor and function efficiently within the tumor (6). Modulation of the tumor microenvironment is of special significance in cancer immunotherapy. However, definitive cancer management presents a major challenge, and it might need a combination of surgical, radiotherapeutic, chemotherapeutic, immunotherapeutic and traditional Chinese medicine therapy approaches. The combination therapy promises to be an effective treatment strategy to address chemotherapy drug resistance by preventing the development of resistance and better than anyone drug alone (7). The introduction of each drug in a combination therapy strategy would depend on the inhibitory pathway that they are in, which involves such questions as the stage of tumor development and which epigenetic drugs should be introduced, when, and in what doses (8). Combination therapy allows the use of corresponding cancer management strategies at each stage of tumor development to achieve optimal results through appropriate treatment modalities. Traditional Chinese medicine has demonstrated excellent antitumor effects in cancer therapy due to its multiple targets, low side effects, and high efficacy (9, 10). It is well known that traditional Chinese medicine is a rich source of natural products, thus, various natural products exhibit low toxic side effects. It has been pointed out that natural products could play an anti-tumor role *via* managing factors in genetics, epigenetics, cancer stem cells, and the tumor microenvironment (11). Therefore, natural products would be a critical composition of combination therapy, making up for the deficiency of surgery, chemotherapy and immunotherapy.

## 2 Natural products possess anti-tumor function

### 2.1 Natural products applicate in tumor

**Figure 2:**
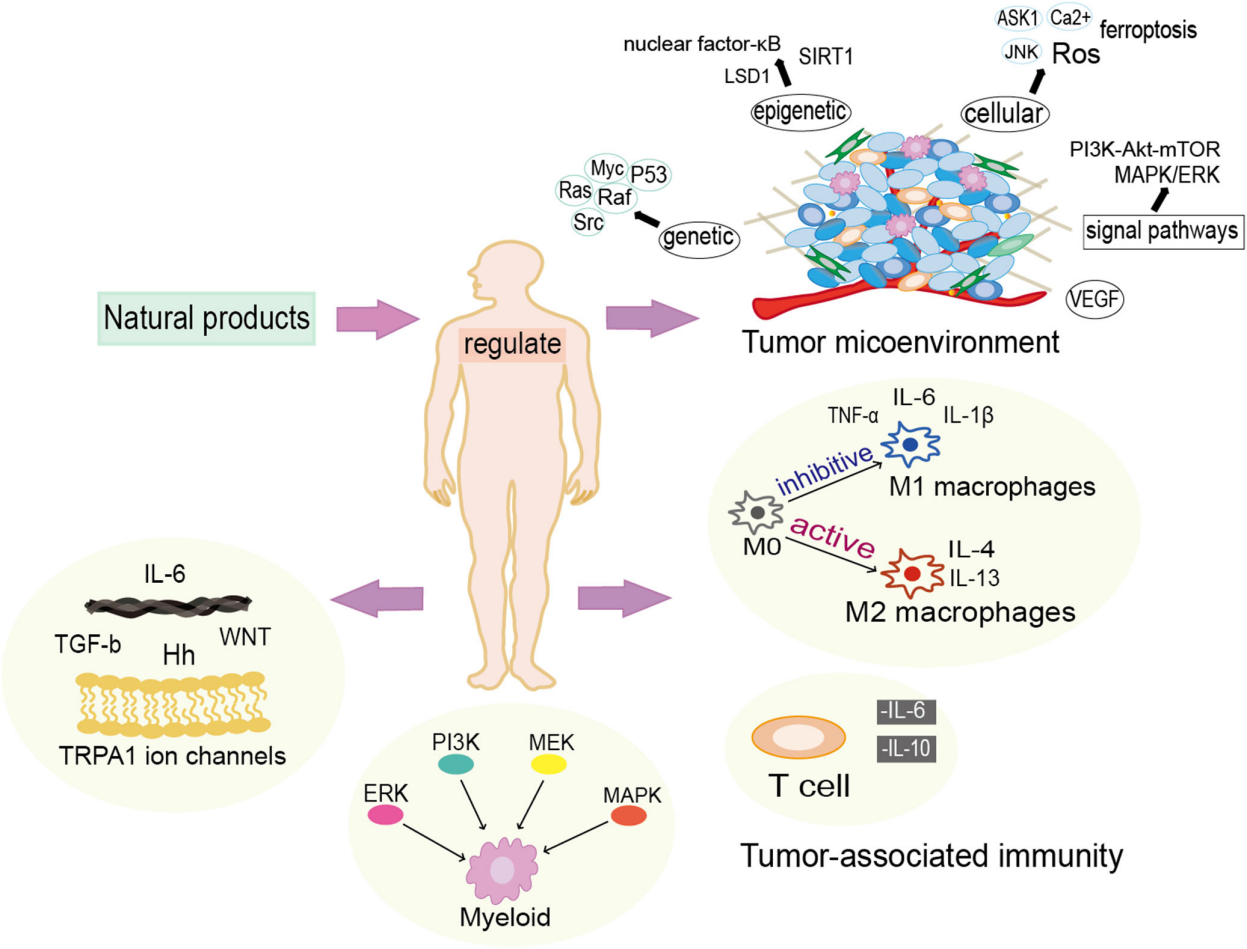
Natural products exert anti-cancer functions in multiple manners. Natural products modulate the tumor microenvironment from genetic, epigenetic and cellular levels, and they also have vital actions in the regulation of inflammatory and immunological responses.

The antitumor effects of natural products have been broadly investigated as well as showed a strong integrative capacity in clinical applications. According to different chemical structure, there are eight major categories of anticancer natural products in usage, including volatile oils, terpenoids, quinone, alkaloids and phenylpropanoids, saponins, flavonoids, isoflavones, polysaccharides and inorganic salts (12). Since natural products always have mild effect, less toxic side effects and multi-targeted features, which are often used for prevention and improvement of symptoms at the early stage of cancer as well as in the treatment of advanced cancer. Natural products have been shown that they could induce apoptosis, anti-proliferation and inhibit metastasis of cancer cells to prevent cancer occurrence and reverse carcinogenesis. However, only a few natural products, such as resveratrol and Epigallocatechin, have been investigated clinically till now (13). Natural products can improve the chemotherapies and contribute to the delivery of nanomedicines to the tumor cells, thus vastly promoting the treatment effects on desmoplastic tumor (14). But in most of the case, natural drugs require structural optimization to improve the efficacy, safety and chemical accessibility as well as pharmacokinetics (15). Generally, natural products could carry out anti-tumor function *via* modulating genetic, epigenetic, cellular and microenvironmental factors (**Figure 2**).

### 2.2 Natural products modulate the expression of cancer associated genes

#### 2.2.1 P53

P53 is a common tumor suppressor gene that regulates cellular senescence, apoptosis, cell cycle arrest and DNA repair, playing an important role in the control of tumorigenesis and progression (16). During the last decade, attentions have been drawn to the possibility of using natural products to control cancer proliferation by utilizing the mechanism of interaction between MDM2 and p53. MDM2 is a major negative regulator of p53. Overexpression of MDM2 is often seen in various human cancers and correlated with high-grade, late-stage tumor (16, 17). Natural drugs have been found to inhibit MDMX (2N0W) and MDM2 (4JGR) proteins to restore p53 protein activity in cancer treatment (18). For example, curcumin effectively upregulates pro-apoptotic proteins (such as p53 and Bax) and downregulates anti-apoptotic proteins (such as MDM2 and Bcl-2) in breast cancer cells (19). Another report shows that curcumin upregulates p53 protein in human colon p53^+/+^HCT116 cells to induce cancer cells apoptosis (20). In colorectal cancer cell lines, resveratrol enhances the expression of p53 and its target genes, such as Bax (21). G2/M phase has been found in berberine-treated human hepatoma cells with enhanced expression of Bax and Apaf-1, activation of caspase 3 and caspase 9, and pro-apoptotic effect by activating the p53 pathway in cancer cells (22). In a natural products intervention trial for skin cancer, dioxin was found to cleave caspase-3/9, significantly increase the expression level of p53, and decrease the expression level of Bcl-2 at the same time (23). Activation of p53 signaling would be contributed to enhance the anti-tumor effect of natural products (**Table 1**).

**Table 1:** Genetic mechanisms underlying natural products-based cancer treatment.

Natural products	gene	Underlying mechanism
Curcumin	H-Ras	Inhibition (24)
	P53	Activation (19)
	RAF/MEK/ERK pathway	Activation (25)
	c-Myc	Inhibition (26)
Resveratrol	H-Ras	Inhibition (27)
	P53	AMPK activation; SIRT1 inhibition (21)
	c-Myc	Inhibition (28)
Quercetin	H-Ras	Inhibition (29)
	RAF/MEK/ERK	Reduce (30)
	c-Myc	Inhibition (31)
Berberine	P53	Activation (22)
	RAF kinases	Inhibition (32)
Dioscin	P53	Activation (23)
Matrine	c-Myc	Inhibition (33)
Myricetin	c-Myc	Inhibition (31)
Piceatannol	c-Myc	Inhibition (31)
Tanshinone IIA	Scr	Inhibition (34)
Shikonin	Scr	Inhibition (35, 36)

#### 2.2.2 Ras

Ras gene has three isoforms (N-Ras, K-Ras and N-Ras), and Ras protein is a cell surface GTP bound protein that is activated by external signals to mediate intracellular signaling pathways (37). The proteins encoded by Ras genes regulate different signaling pathways in malignant cells that are important for the development and progression of cancer cells, including angiogenesis, cell survival, proliferation, cell cycle distribution, and migration (38). Various studies have confirmed that natural products can enhance the treatment effect of cancer by inhibiting Ras gene expression and regulating Ras signaling pathway. For example, quercetin is found to enhance the sensitivity of cisplatin by modulating the miR-217-KRAS axis, suggesting that quercetin in combination with cisplatin can be used to improve the treatment of osteosarcoma (39). Piperlongumine effectively prevents colon cancer by targeting Ras protein and PI3K/Akt signaling cascade to suppress the activity of Akt/NF-κB, c-Myc and cell cycle protein D1 (40). It is well known that PI3K-Akt-mTOR signaling pathway plays an important role in various type of cancer cells (41). It has been confirmed that curcumin arrests cancer cells in G2/M phase by potentiating Erk1/2 and inhibiting Akt together with its corresponding downstream molecules (mTOR and S6K1) in Ras-activated HAG-1 human adenocarcinoma cells (24). Resveratrol is one of the common natural drugs used in cancer treatment. Previous studies have demonstrated that resveratrol can inhibit EGFR phosphorylation and subsequent activation of Ras/Rho/ROCK signaling to combat the invasive proliferation of ovarian cancer cells (27). The above information confirms that Ras signaling might center in many natural products-based cancer treatment.

#### 2.2.3 Raf

Raf contains three subtypes: A-RAF, B-RAF and C-RAF, of which, the mutations in B-RAF are mainly correlated with carcinogenesis. B-RAF (B-RAF V600E) mutations have been suggested to be biomarkers for diagnosis and prediction of many cancers, including colorectal, thyroid and melanoma (42). Statistically, B-RAF mutations account for 66% of melanomas and 7% of all cancers (43). The genetic alterations that aberrantly activate RAS/RAF/MEK/ERK signaling mainly occur within or upstream of RAF. Nowadays, a breakthrough has been made in the development of RAF inhibitors to treat B-RAF (V600E) mutated cancers (44, 45). However, resistance to RAF inhibitors has emerged in subsequent clinical studies, thus researches are trying to look for solutions from natural products. B-RAF activates C-RAF and develops resistance to B-RAF inhibitors, three berberine derivatives (BBR-7, BBR-9 and BBR-10) are available as lead compounds against RAF kinases (43). Besides conquering resistance to RAF inhibitors, natural products might also prevent cancer development *via* modulating Raf expression. Curcumin has been found to regulate cellular autophagy to trigger anti-leukemic mechanism or activate the RAF/MEK/ERK pathway to induce autophagic death of early SUP-B15 cells (25). Quercetin could prevent prostate cancer by reducing the production of androgen receptor in the RAF/MEK/ERK pathway (30). Targeting Raf might be helpful to improve the application of natural products in cancer treatment.

#### 2.2.4 Myc

Myc is a common nuclear transcription factor with three types, c-Myc, n-Myc, and l-Myc. Among them, c-Myc is always considered as an important target for cancer therapy (46, 47). Multiple oncogenes can exert carcinogenic effects by upregulating the expression of c-Myc (48, 49). Myc possesses a widespread regulatory function, embracing cell metabolism, metastasis, proliferation, differentiation and apoptosis (48). Overexpression of c-Myc contributes to the growth and migration of prostate cancer (PC) cells, as well as causing a series of resistance to radiotherapy and chemotherapy in cancer cells (50). Research related to natural drug treatment of T-cell lymphoma (NKTCL) reveals that matrine treatment effectively reduces c-Myc gene expression and accelerates the degradation of c-Myc protein (33). In addition, curcumin similarly inhibits the proliferation of gastric cancer cells by reducing the expression of the c-Myc oncogene (51). Last year, some scholars conducted an experimental of curcumin analogs targeting the formation of G-quadruplex structure in human c-Myc gene promoter against tumor by using MCTS model which microenvironment is similar to vascular tumor, revealing a new c-Myc -mediated curcumin anti-cancer pathway (52). Resveratrol suppresses the c-Myc/miR-17 pathway in breast cancer cells to upregulate major histocompatibility complex class I chain-related proteins A and B (MICA and MICB) that promote antitumor immune responses by increasing cytolysis of breast cancer cells by natural killer (NK) cells (28). A significant downregulation of c-Myc in medulloblastoma mediated by resveratrol has been found to be intimately associated with apoptosis, growth inhibition and cell cycle arrest in medulloblastoma cells (53). Several natural products, including resveratrol, myricetin, piceatannol and quercetin, could upregulate HIF-1α by activating SIRT1, thereby downregulating the expression of c-Myc, PHD2 and β-linked protein (31). These natural products could be potential inhibitors for c-Myc.

#### 2.2.5 Src

Src gene family also owns multiple types, and Src family kinase (SFKs) acts as a cytoplasmic tyrosine kinase involved in diverse intracellular signaling cascades, in turn affecting cell differentiation and migration (54, 55). Over-activation of Src occurs in many human cancers. It has been demonstrated that Src kinases initiate multiple signaling cascades in the tumor microenvironment (TME), leading to the occurrences of tumor growth, angiogenesis, migration and drug resistance (55, 56). Therefore, investigation on Scr inhibitor may provide a more precise and efficient therapeutic pathway for cancer treatment. Natural product Tanshinone IIA could achieve anti-osteosarcoma cancer cell effect by blocking the progression in downstream of MAPK/ERK and PI3K/AKt signaling pathways through inhibition of Src kinase (34). Shikonin stunts metastasis of human ovarian cancer cells by inhibiting Src and FAK protein tyrosine kinases (35, 36). β-hydroxyisopentylshikonin, a derivative of shikonin, has been found to inhibit v-Src receptor protein tyrosine kinase (PTK) activity (35, 36). Natural products treatment in combination with Src prevention would be an alternative strategy for cancer therapy.

### 2.3 Natural products modulate epigenetics

Epigenetic alterations generally arise in various human cancer cells, in which normal cells are transformed into cancer cells after abnormal epigenetic alternations in critical genes that are associated with cancer (57, 58). Common epigenetic modifications mainly include histone methylation, histone acetylation, DNA methylation, microRNAs and alternative splicing. Additionally, there are some ways, such as demethylation of oncogenes or cancer-promoting genes (CpG), to upregulate gene expression through epigenetic de-repression mechanisms (58). Epigenetic mechanisms are shaped by a series of intricate crosstalk and can form distinct epigenomic profiles depending on different microenvironmental contexts, therefore, epigenetic inheritance varies across tumors (59). Polycomb group (PcG) proteins have two complex types belonging to the family of epigenetic modifiers, of which Polycomb repressive complex 1 (PRC1) is a histone ubiquitin ligase and PRC2 is a histone methyltransferase (60, 61). Misregulation of multiple comb proteins in distinct cancers leads to different results, while pan-cancer analyses indicate that epigenetic factors seem to be the primary contributor to tumorigenesis (62). As we all known, epigenetic modifications primarily occur in DNA and histone proteins, but there is a growing awareness that epigenetically modified non-coding RNAs may be the target of new therapeutic pathways for cancer (63, 64). Non-coding RNAs play an essential part in the control of epigenetic mechanisms, such as long non-coding RNAs, microRNAs and piwi-interacting RNAs (63).

Alterations in epigenetic states can be potential targets for cancer therapy, and many studies have demonstrated the possibility of epigenetic inhibitors and natural epigenetic regulatory substances to alter abnormal epigenetic states thereby inhibiting cancer progression (65). Histone lysine specific demethylase 1 (LSD1) is the basement for the development of many pathologies, and research has shown that a number of natural products such as resveratrol, curcumin, protoberberine, stilbene, diterpenoids, and flavones have an inhibitory effect on LSD1 (66). These natural products are effective chemotherapeutic agents for treating cancer through a variety of pathways and processes, including epigenetic mechanisms that alter the capacity of cancer cells in different ways (67). For example, curcumin exerts powerful anti-inflammatory response, anti-oxidative stress and anti-cancer capacity by regulating histone modifications, DNA methylation, nuclear factor-κB and nuclear factor erythroid-2-related factor 2 of epigenetic signal pathways (68). Epigenetic changes in DNA methylation, histone modifications and expression of miRNAs in resveratrol and pterostilbene have been examined, and results revealed that both natural products activated SIRT1 to enhance therapeutic efficacy as well as restore hypermethylation and hypomethylation of critical oncogenes and suppressors in tumors (69). Therefore, an in-depth study of the relationship between natural products and epigenetic modifications in cancer cells might further enhance the potential for effective cancer treatment at the epigenetic level.

### 2.4 Natural products maintain cellular homeostasis

Cellular homeostasis mechanism, including Ca^2+^ homeostasis, proteostasis, and redox homeostasis, contribute to maintain an internal stability in response to environmental disturbances. Redox regulation is intimately associated with tumorigenesis, tumor microenvironment, cellular autophagy, programmed cell death, and metabolic reprogramming, which promote cancer progression through multiple regulatory effects (70). Cancer cells tend to possess high levels of adaptive antioxidant system and highly reactive oxygen species (ROS), and increased levels of reactive oxygen species disrupt the initial redox homeostasis, which promotes tumor proliferation, metastasis and drug resistance (71, 72). Although high ROS levels can induce apoptosis of cancer cells, their prolonged activation inevitably reduces the efficacy of chemotherapy due to the establishment of drug resistance (73, 74). Combination therapies to combat chemotherapy resistance have been extensively studied in clinical trials, while cancer treatment strategies that modulate ROS to modify the tumor microenvironment urgently need more in-depth research (75). Research has shown that oxidative stress is increased in T cells and decreased in mucous cells in the gastric cancer microenvironment, while TRIM62, MET and HBA1, which are associated with oxidative stress, may be biomarkers reflecting the prognosis of gastric cancer (76). Selective targeting of ROS-mediated signal pathways, including the role of tracer elements, systems regulated by the stress response transcription factor Nrf2 and nuclear factor-κB, as well as environmental factors, may increase the precision of the redox agents used (77).

ROS are the key to anti-cancer effect of natural products, therefore, natural products with multi-targets and few adverse effects in cancer therapy have attracted widespread attention (78). Radiation causes cells to produce ROS. Radiation studies have found that epicatechin, silibinin, genistein and apigenin function as antioxidants to scavenge free radicals and anti-inflammatory agents (79). Nrf2 controls the expression of detoxification enzymes and antioxidants. Therefore, it is possible that natural products capable of suppressing the Nrf2 and NF-κB pathways are more desirable agents for radiotherapy chemotherapy (80). Curcumin has been demonstrated to trigger the rise of ROS in tumor-associated fibroblasts, which causes endoplasmic reticulum stress *via* the PERK-eIF2α-ATF4 axis, thus leading to cell cycle arrest and apoptosis (81). At the same time, curcumin has been shown to increase ROS accumulation, which promotes cellular senescence and apoptosis in human cervical cancer cell lines after treatment with reactive oxygen species scavengers (82). The results suggest that curcumin has good effects in raising ROS levels to stimulate apoptosis in cancer cells. In clinical trials of natural products combined with cisplatin for the treatment of melanoma, berberine acted more like a chemosensitizer, effectively activating the ROS/p38/caspase cascade that stimulates melanoma cell death (83). Moreover, berberine triggers apoptosis-related responses such as cleavage of caspase-3, release of cytochrome c and depolarization of the mitochondrial membrane in non-small cell lung cancer through activation of apoptosis signal-regulated kinase 1 (ASK1) and c-jun NH2 kinase (JNK) *via* the ROS pathway (84). Natural products further enhance the therapeutic efficacy of cancer treatment through regulating intracellular ROS levels *via* multiple signal pathways, to switch on the cell death cascade.

Ferroptosis is a mode of immune cell death. The occurrence of immune cells ferroptosis in the tumor microenvironment is also intimately correlated with ROS levels. Excessive accumulation of intracellular lipid reactive oxygen species (ROS) triggers iron sagging-mediated cellular programmed death (85). ROS-induced autophagy has been found to have a critical regulatory function on both TfR1 expression during ferroptosis and ferritin degradation (86). Iron not only provokes ferroptosis-induced cell death after raising intracellular ROS levels, but also that it is able to trigger pyroptosis death through Tom20-Bax-caspase-GSDME pathway (87). In addition, flavonoids such as quercetin and anthocyanin have been found to alter intracellular Ca^2+^ homeostasis by regulating plasma membrane Ca^2+^-ATPase (88). Studies indicate that iron may cause ferroptotic neuronal cell death through activation the redox-sensitive Ca^2+^ channel to disrupt Ca^2+^ homeostasis (89). Persistent endoplasmic reticulum stress causes the accumulation of unfolded proteins in the endoplasmic reticulum, which leads to cell death. Natural products are able to exert positive anti-cancer effects on gastric, lung, colorectal, breast and liver cancers by regulating the endoplasmic reticulum stress (90). Therefore, exploiting the ability of natural products to regulate redox homeostasis, Ca^2+^ homeostasis, and protein homeostasis to impact cell viability may become a cancer therapeutic means to promote cancer cell apoptosis.

### 2.5 Natural products regulate tumor microenvironment

Cancer possesses a complex heterotypic tumor microenvironment. Tumorigenesis and progression are not only related to cancer cells, but also intimately linked to the dynamic processes of the tumor microenvironment. As shown in **Figure 1**, the basic constituents of the tumor microenvironment are neuroendocrine cells, adipose cells, immune cells, inflammatory cells, myofibroblasts and fibroblasts, the blood and lymphatic vascular networks, and extracellular matrix (91). Each type of these cells has its own unique immunity, but their antitumor function is reduced in the tumor microenvironment, and the mechanism of sustained activation of the NF-κB pathway generation also appears to favor tumor survival and drive abortive activation of immune cells (92). The tumor microenvironment is frequently accompanied with inflammatory and immune responses. In order to inhibit cancer cell migration and proliferation by remodeling the tumor microenvironment, a large number of studies have been conducted to investigate the influencing factors of tumor microenvironment. The immune activation response of immune cells induces tumor cells to secrete cytokines, and the dynamic variation of cytokines in turn determines the differentiation of immune cells. For example, the coordination between long non-coding RNAs with immune cells and cytokines could accomplish the remodeling of tumor microenvironment (93). The changing state of the tumor microenvironment reflects the process of tumor deterioration. Therefore, tumor viability can be rapidly regulated by intervening in the surrounding environment which tumors directly contact.

As described above, the tumor microenvironment is a critical determinant in tumorigenesis and proliferation. Changes in the tumor microenvironment are frequently followed by immune and inflammatory responses, which are intimately linked to the remodeling of the tumor microenvironment. Components of tumor which perform immune functions also known as the tumor immune microenvironment (TIME) are inseparably associated with tumorigenesis, metastasis and problems of poor cancer prognosis (94). Initially, immune cells in the tumor microenvironment are able to kill cancer cells through immunomodulatory effects, but cancer cells gradually escape the immune cell surveillance system as cancer progresses, even through various inhibitory mechanisms to antagonize the toxic effects of immune cells (95). Long non-coding ribonucleic acids (lncRNAs) impact the occurrence and the immune response of cancer cells in the TIME through a variety of regulatory mechanisms (96). The functional analysis of lncRNA thus helps to increase the level of immune stimulation in the tumor microenvironment, enabling the immune system to eliminate cancer cells from the body (97). Bone marrow cells, cancer-associated fibroblasts, tumor-infiltrating neutrophils, and tumor-associated macrophages are critical components of the gastric cancer tumor microenvironment that ultimately dictate the direction of tumor development according to genetic and epigenetic factors (98).

Inflammation is an important hallmark of cancer, while the systemic inflammatory response and the local immune response are inextricably linked to the development of tumors and the survival of patients with cancer (99). Numerous clinical trials have demonstrated that chronic inflammation correlates with the occurrence of multiple cancers and that solid tumors also initiate the local inflammatory system to foster cancer cells proliferation and metastasis (100). Chronic inflammation is caused by the action of immune cells such as neutrophils, macrophages and eosinophils or pro-inflammatory cytokines, which initiate a sustained active inflammatory response and varying degrees of tissue damage (101). Damage to intracellular DNA and other macromolecules by the inflammatory response leads to DNA methylation and microRNAs dysregulation, while a vicious cycle of oxidative stress occurs (102). The inflammatory microenvironment is an essential component of the tumor microenvironment, where cytokines such as VEGF-A and CCL2/MCP-1 induce angiogenesis with the recruitment of monocytes and neutrophils into the tumor niche to transform into tumor-associated monocyte-macrophages (TAM) and tumor-associated neutrophils (TAN) (103, 104). At the same time, inflammatory response is tightly related to the regulation of tumor microenvironment and implementation of cancer immunotherapy. Studies have indicated that the immune phenotype associated with human tumor microenvironment is also presented in non-malignant inflammatory tissues (105). Therefore, specific studies targeting immune changes at the site of inflammatory response in the tumor microenvironment are also an option for cancer treatment. Cancer stem cells are associated with the tumor microenvironment which enable their evolution and even complete transformation into malignant cells under complex genetic and epigenetic effects (106). Modulating cancer stem cell development-associated Hedgehog, WNT, Hippo, and Notch signal pathways by inhibiting the immune mediators in the tumor microenvironment is a critical means for cancer immunotherapy (107).

Changes in tumor microenvironment are likely to lead to subsequent changes in the development of cancer cells, therefore, searching for highly targeted and less toxic therapy drugs to moderate tumor microenvironment is a feasible option for cancer treatment. For instance, carotenoids, flavonoids and polyphenols and other natural products may combat cancer by engaging in epigenetic modifications, cell signaling and cellular responses (108). In addition, combined treatment strategy of natural products with immunosuppressive agents has been shown to be more effective in halting lung cancer than monotherapy in trials of natural anticancer products for lung cancer tumor microenvironment (109). Colon cancer (CRC) clinic research has found that natural products may affect the NK cells, T cells and Tregs of CRC to perform immunomodulatory functions for anti-tumor (110). Natural products have been shown to restore immune-suppressed tumor microenvironments under a single target and organ-specific inflammatory strategy, recruiting subpopulations of immune cells to perform their functions in the microenvironment (111). Natural products also have significant actions on tumor immunosuppressive factors and cells, promoting the secretion of anti-tumor immune factors (IL-1βI, TNF-α, FN-γ), inhibiting the secretion of immunosuppressive factors (TGF-β, IL-10, PGE2), down-regulating the number of immunosuppressive cells (MDSC, Tregs, M1 macrophages) and enhancing the TEFF activity and quantity (112). Natural products promote cancer immunotherapy by influencing immune actions of critical cell populations in the tumor microenvironment, including fibroblasts, inflammatory factors, mast cells, and macrophages, thereby eliminating cancer cells.

## 3 Mechanisms underlying natural products remodeling tumor microenvironment: immune cells and inflammatory cytokines

### 3.1 T cells

T cells, are crucial lymphocytes in the body, dominate the inflammatory response, wound healing and tumor immune surveillance processes. T cells modulate other types of immune cells by producing various cytokines in cellular immune responses, which in turn elicit further immune actions (113). The capacity for infiltration and suppression of tumors varies between the different types of T cell subsets (114). According to previous studies, CD4^+^ T cells differentiate into T helper 1 (Th1), Th2, Th17, and T follicular helper (Tfh) effector T cell subsets under different cytokine expression patterns, while CD8^+^ T cells also differentiate into T helper 1 (Th1), Th2 and Th17 subsets based on cytokine expression types (113, 114). During recent years, the advantages of immunotherapy for cancer treatment have attracted considerable attention from academics. Immunotherapy usually has excellent efficacy both in addressing chemotherapy resistance and in improving the survivability of patients with mid-to-late-stage cancer. Unfortunately, solid tumors generate an immunosuppressive tumor microenvironment that makes cancer antigens poorly delivered to immune cells, evading anti-cancer immunity (115). Therefore, screening for new targets in different tumors, selecting appropriate tumor biomarkers, and combining conventional therapeutic with immunotherapeutic modalities may be superior options for improving clinical outcomes in cancer immunotherapy (116).

The specificity for tumor-expressed antigens of T cells provides an important contribution in cancer immunotherapy, while the longevity, functionality and durability of effector T cells also determine the results of immunotherapy (117). According to a previous study, single-cell RNA sequencing (scRNA-seq) analysis of more than 50,000 resting and activated T cells revealed that the activation level of CD8^+^ T cells is stronger than CD4^+^ T cells in most tumors (118). Tumor-infiltrating NKG2A^+^CD8^+^ T cells are the predominant lymphocyte subset in lung cancer, and the expression of NKG2A on tumor-infiltrating CD8^+^ T cells also regulated by TCR, thus NKG2A^+^CD8^+^ T cells may offer an efficient solution for immunotherapy for lung cancer (119). Urologic cancer patients have increased levels of Th2 cytokines in double positive (DP) CD4^+^ CD8^+^ T cells, while both CD8α- and CD8α-β-DP T cells induce Th2 polarization of naive CD4 T cells as well as suppress Th1 activation (120). Tumor-reactive T cells (pTRT) vary in different tumor microenvironments, with CD8^+^ T cells mainly switching in natural killer (NK)-like T cells and terminal exhausted T cells, CD8^+^ Treg cells, and type 17 CD8^+^ T cells (121). Disturbances from T cells internal and external factors in the tumor microenvironment as well as persistent TCR triggering may trigger T cell dysfunction, moreover, immunosuppressive responses in the tumor microenvironment may further exacerbate T cell dysfunction (122).

Natural products have incalculable potential in the treatment of cancer. Natural products regulate the immunosuppression level in the tumor microenvironment by modulating T-cells status aiming at immunotherapy of cancer (**Table 2**). A study of natural products regulating immune cell function in advanced colorectal carcinoma (CCA) found that regulatory T cells (Treg) in the CCA tumor microenvironment were successfully converted to T helper 1 (Th1) cells by curcumin (129). These results have been reinforced in another lung cancer study, in which curcumin increased interferon expression and inhibited forkhead protein-3 gene transcription to convert Tregs into Th1 cells, suggesting that curcumin may have anti-tumor effects by modulating Tregs cells to affect tumor-specific immune tolerance (133). In head and neck squamous cell carcinoma (HNSCC), curcumin increased tumor infiltrating lymphocytes (TILs) and decreased the expression of mucus structural domain 3 (TIM-3) cells and programmed cell death protein 1 (PD-1), which enhances the possibility of targeted therapy for HNSCC (131). Berberine has been shown to inhibit the activation of regulatory T cells (Tregs) and attenuate the activity of immunosuppressive myeloid-derived suppressor cells (MDSCs) as well as enhance tumor-infiltrating T-cell immunity resulting in antitumor effects (134). Besides, berberine inhibits tumor antigen-mediated IL-6 and TGF-β expression as well as IL-10 proliferation but restores anti-tumor cytotoxicity of T cells in the tumor microenvironment (135). Th1 immune responses were intensified and CD8+ T cell activity was significantly enhanced after resveratrol treatment of mouse lung tumors, which may be due to the downregulation of PD-1 expression on CD4^+^ T cells and CD8^+^ T cells in lungs of tested mice (136). Wogonin and Scutellaria ocmulgee leaf extract (SocL) both inhibit TGFβ1 secretion and reduce TGFβ1 induced Treg activity in malignant gliomas, thereby reversing tumor-mediated immunosuppression (134). Natural products are generally versatile active substances that can directly or indirectly modulate the immune response by affecting the activity of T-cell groups, which in turn suppress or even eliminate cancer cells.

**Table 2:** Natural products participate in the regulation of T cells.

Type of T cells	Natural products	Underlying mechanism
Tregs	Curcumin	Inhibition (123)
Th1	Curcumin	Activation (124)
TILs	Curcumin	Increase (125)
tumor-infiltrating T-cell	Berberine	Activation (126)
Tregs	Berberine	Inhibition (126)
CD8^+^T cell	Resveratrol	Activation (127)
Th1	Resveratrol	Increase (127)
Treg	Wogonin	Inhibition (128)
Treg	Scutellaria ocmulgee leaf	Inhibition (128)
Th17	Dioscin	Inhibition (129)
Treg	Dioscin	Activation (129)
Treg	Baicalein	Activation (130)
Treg	Shikonin	Activation (131)
CD4^+^T cell	Osthole	Activation (132)
CD8^+^T cell	Osthole	Activation (132)

### 3.2 Myeloid-derived suppressor cells

Myeloid-derived suppressor cells are immature heterogeneous myeloid cells population, which have immunosuppressive function in reducing the activity of natural killer cells and suppressing the proliferation of T cells (137). Myeloid-derived suppressor cells (MDSC) are divided into two groups, monocytes (M-MDSC) and multinucleated cells (PMN-MDSC), with M-MDSC showing a remarkable plasticity and the direction of differentiation regulated by the tumor microenvironment (138). Normal tissues utilize myeloid cells as important participants in the organism’s defense to pathogenic infections and in the completion of tissue remodeling, but in abnormal tumor tissues MDSC have taken on this task (139). The main reason lies on that the sustained myelopoiesis occurs in autoimmune diseases, chronic inflammation or cancer, resulting in a suppression mechanism of B-cell, T-cell and NK-cell functions, which eventually deviates the standard differentiation pathway leading to the pathological activation (140, 141). Tumor emergence is inevitably associated with inflammation and immune response, so myeloid-derived suppressor cells perform an important function in tumor formulation and progression. The increased number of myeloid-derived suppressor cells in cancer patients, which have a significant function in promoting angiogenesis, cancer cell proliferation and metastasis, probably results from extended survival and increased viability of myeloid-derived suppressor cells induced by autophagy regulation by high-mobility group box protein 1 (137, 142). MDSCs have now become an inevitable factor influencing tumor immune responses and it is urgent to identify biomarkers that regulate this cell population to control the direction of MDSCs. In the complex tumor microenvironment, MDSC have a series of interactions with cancer cells. Therefore, shaping the tumor immune microenvironment through targeted therapy or mild natural products to regulate MDSC may be a better therapeutic method for cancer. Research in the emerging field of immunometabolism has revealed that intratumoral myeloid cells would readjust their cell metabolic profile for adaptation of the nutrient-limited tumor microenvironment, hence providing a great immunotherapeutic value by means of cell metabolically reprogramming (143).

Among the breast cancers, the breast cancer cells recruit MDSC using cytokines and chemokines *via* three pathways, STAT3/IRF-8, PTEN/Akt and STAT3-NF-κB-IDO, to suppress anti-tumor immune responses and facilitate cancer cell proliferation (144). In another study, myeloid-derived suppressor cells (MDSC) inhibited T-cell function under signal transducer or activator of transcription 3 (STAT3) activated conditions, eventually generating a negative immunomodulatory response to aid tumor resistance to immune defenses (145). The remarkable immunosuppressive effect of MDSC in tumors blocks the immunotherapeutic effect of target programmed cell death protein 1 (PD-1), but the cruciferous product 3,3 ‘-diindolylmethane (DIM) can promote T cell responses to enhance the therapeutic effect of PD-1 antibodies, thus arresting cancer cell proliferation (146). Natural products have better immunomodulatory functions in the tumor microenvironment, and they also have great potential in targeting MDSC to inhibit cancer cells. Research has demonstrated that berberine promotes the proliferation of granulocyte-myeloid-derived suppressor cell (G-MDSC)-like population by activating IL6/STAT3 signal pathway, furthermore, berberine significantly upregulates the transcriptional levels of Sox2 and Oct-4 to enhance the transformation efficiency of G-MDSC-like population (147). Curcumin remarkably inhibits the expression of arginase-1 (Arg-1) and ROS of MDSC in tumor tissues, and also decreases IL-6 in serum and tumor tissues of LLC-bearing mice to prevent the multiplication of MDSC (148). Curcumin suppressed the expression of granulocyte-colony stimulating factor (G-CSF), granulocyte-macrophage colony-stimulating factor (GM-CSF), the TLR4/NF-κB signal pathway and inflammatory factors in liver cancer, causing a dramatic reduction of MDSC cell numbers in tumors (149). However, quercetin, a natural product frequently utilized in cancer therapies, enhances the survival of human-derived G-MDSC while also contributing to the secretion of T-cell suppressors *in vitro* (145). In conclusion, the modulation of MDSC by various natural products in different cancers should be adequately investigated to get a more accurate cancer immunotherapy regimen.

### 3.3 Mast cells

In cancer clinical therapy, the stages of tumor progression and prognostic effect always demand multidimensional target monitoring. Mast cells, which are a type of tissue-resident immune cells of the bone marrow lineage, play an important role in regulating cancer cell characteristics and anti-tumor immune responses, with enormous value of cancer immunotherapy investigations (150). Mast cells work as both positive and negative regulators of the immune response which can exert a tumor-promoting or repelling effect on cancer cells directly or through tumor microenvironment and immune response indirectly (151). Mast cells have been shown to be potentially critical coordinators of the initial phase of the antitumor immune response in cancer therapy, capable of treating cancer through mast cell stabilizers, mast cell mediator modulators, anti-targeting inhibitory receptors and ligands, FϵR1 signal pathway activators/inhibitors, TLR activators and c-KIT inhibitors (150). Mast cells can modify or even reverse the suppressive properties of Treg cells which are considered as an important constituent in the tumor microenvironment as a central component in controlling innate and adaptive immunity, a novel target to improve cancer immunotherapy efficacy (151, 152). Surprisingly, as a type of heterogeneous immune cell, mast cells showed contrasting effects in different types or in specific development stages of cancer. Analogous to a double-edged sword, mast cells have both pro-angiogenic effects to support cancer development and anti-tumor effects in certain types of cancer (153). For example, in breast cancer, mast cells may serve as functions of promoting tumor progression to produce poor prognosis and inhibiting cancer cell viability to improve cancer treatment outcomes. On the one hand, mast cells release proteases and pro-angiogenic factors to activate sequestered growth factors in extracellular matrix, further promoting fibroblast multiplication and degrading extracellular matrix, eventually accelerating cancer cell proliferation and metastasis (154). On the other hand, mast cells could assist breast cancer immunotherapy by secreting toxic cytokines, selectively inhibiting tissue remodeling and angiogenesis, as well as prohibiting auto-mediated immunosuppressive responses (155). In conclusion, the regulatory of mast cells on tumors is complicated, but regulating mast cell actions by agents is a feasible option for safe tumor treatment.

Over the recent years, with the potential of mast cells being explored in cancer immunotherapy, extensive studies revealed that natural products could regulate the function of mast cells. Paclitaxel exerts antitumor effects in non-small cell lung cancer, breast cancer, ovarian cancer and several other cancers (156). But patients frequently suffer serious hypersensitivity reactions and painful sensory neuropathy during the paclitaxel chemotherapy (157). The occurrences of adverse reactions may be associate with paclitaxel activation mast cells by the following mechanisms: complement activation, non-IgE-mediated idiosyncratic mast cell degranulation by paclitaxel, and IgE-mediated mast cell degranulation induced by paclitaxel (156). A study reveals that quercetin has the potential to improve paclitaxel-induced pain produced by neuropathy (157). An investigation of mast cell degranulation sensitization mechanisms shows that quercetin can inhibit mast cell allergic by activating Nrf2-HO-1 signal pathway (158). Quercetin functions as an anti-inflammatory, anti-oxidant and anti-cancer agent by interacting with such molecules as mitogen-activated protein kinase (MAPK), phosphatidylinositol-3-phosphate kinase (PI3K), extracellular signal-regulated kinase (ERK) and kinase (MEK)1 (159). The above information suggest that quercetin has an important contribution in regulating immune responses involved in mast cells as well as in controlling tumor proliferation. Indeed, quercetin inhibits the cross-linking of FceRI and other activating receptors on mast cells, also reduces the available of nitrite and affects vascular function thus inhibiting tumor metastasis and proliferation (159). In addition, curcumin has been shown to inhibit mast cell activation, which is mediated by blocking the ERK pathway to inhibit protease-activated receptor 2 (PAR2) and protease-activated receptor 4 (PAR4) (160). These studies prove that natural products are able to regulate the anti-inflammatory and anti-tumor effects of immune cells in the perspective of mast cells.

### 3.4 Tumor-associated macrophages

Tumor-associated macrophages (TAMs) are ubiquitous infiltrating immune cells within cancer tissue. Resembling the dual function of mast cells, TAMs are classified in two functional types. Of these, the activated M1 macrophages eliminate cancer cells by direct cytotoxic effect and indirect cytotoxic (ADCC) effect, while alternatively activated M2 macrophages facilitate cancer cell invasion and metastasis by promoting angiogenesis and suppressing immune cell anti-tumor function (161). There is a mutual interaction between TAMs and the tumor microenvironment. Metabolites derived from immune cells, tumor cells and stromal cells in tumor microenvironment can regulate the metabolic response of TAMs, and the cellular products of TAMs also affect the survival and progression of tumors (162). TAMs are the primary immune cell population in the tumor microenvironment with the type of activation usually determining the fate of tumor development. For example, TAMs are susceptible to polarization to the M2 type in response to colony-stimulating factor-1, M2 macrophages secrete chemokines, cytokines, exosomes and enzymes which in turn triggers cancer survival signal pathways to promote ovarian cancer cell survival and metastasis (163). Obviously, M2 macrophages effectively enhance the viability of cancer cells in the tumor microenvironment and exert immunosuppressive functions to protect them, which directly contributes to the severe deterioration and poor prognosis of ovarian cancer. This study found that the deubiquitinating enzyme (DUB) gene was highly expressed in M2 macrophages from lung cancer tissues, suggesting that reprogramming may effectively regulate the adaptive immune system of cancer immunotherapy and kill tumor cells (164). TAMs have a key function in the tumor microenvironment of many cancers, with macrophages regulating tumor cell growth by a variety of mechanisms, including inflammatory responses, infiltration and cytokine interactions. Through in-depth research on colorectal cancer-associated macrophages, it discovers that Elk-1 expression is positively related to inhibitory receptor signaling regulatory protein-α (Sirpα) expression, which may be intimately connected with the difficulties in curing colorectal cancer malignancies (165). Adequate analysis of the mechanisms underlying the action of tumor-associated macrophages in CRC and complementary macrophage immunotherapy with immune checkpoint inhibitors (CTLA-4 antibodies, anti-PD-1 and PD-L1) can strengthen the treatment outcome (166). It should conduct more detailed classification of TAMs in different cancer types, which will provide a functional analysis of heterogeneous TAMs cells in different tumor microenvironments for accurate treatment *via* therapeutic targeting.

Previous studies have shown that natural products may be associate with the polarization of heterogeneous macrophages, thus clarifying the molecular regulatory mechanisms of natural products within macrophages could provide novel solutions for cancer immunotherapy (167). TAMs are found in many types of cancer, including lung, ovarian and breast cancers, of which M2 macrophages are most prone to cause malignant tumors. Various natural products such as lignans, terpenoids, flavonoids, alkaloids, coumarins and polyphenols are capable to regulate M2 macrophage polarization response through several different molecular mechanisms (168). In other words, if the natural products can cause some changes in the direction of M2 macrophage polarization, they can regulate immunosuppressive effects to kill cancer cells. Quercetin in F4/80^+^/CD11b^+^/CD86^+^ macrophages not only inhibit macrophage polarization but also decrease interstitial fibrosis and extracellular matrix over-accumulation *via* antagonizing TGF-β1/Smad2/3 signaling (169). Quercetin effectively decreases the expression levels of tumor necrosis factor (TNF)-α, interleukin (IL)-6 and IL-1β in M1 macrophages and increases the expression levels of glutamate-cysteine ligase catalytic subunit (GCLC), heme oxygenase (HO)-1 and glutamate-cysteine ligase modifier subunit (GCLM) in M2 macrophages (170). Experiments further confirm that quercetin has a dual regulatory function in tumor-associated macrophages, namely inhibiting the polarization of M1 macrophages and stimulating the polarization of M2 macrophages. Curcumin also has similar functions, avoiding M1 macrophage polarization by inhibiting macrophage-inducible C-type lectin as well as inducing M2 macrophage polarization by promoting IL-4 and IL-13 gene expression (171, 172). M2 macrophages inhibit the induction of vascular endothelial cell growth factor after resveratrol treatment thereby affecting the invasion and migration of human lymphatic endothelial cells as well as effectively inhibiting lymphangiogenesis (173). Series of experiments proved that natural products have a significant contribution in regulating the activity of TAMs to suppress cancer cell proliferation and enhance immunotherapy efficacy.

### 3.5 Cancer-associated fibroblasts

Cancer-associated fibroblasts (CAFs) have high plasticity and frequently present in various kinds of tumor tissues. CAFs are capable of both producing the essential constituents of the cancer tumor microenvironment and releasing regulatory factors to promote cancer progression in the tumor microenvironment. Study shows that CAFs modulate the surrounding tumor microenvironment by synthesizing and remodeling the extracellular matrix as well as by releasing various cytokines, which in turn influence the important cancer cell processes of dormancy, growth and migration (174, 175). Indeed, CAFs refine the structure and function of the tumor stroma by producing extracellular matrix components, and also produce metabolites, cytokines and exosomes that affect tumor metabolism, immunology and angiogenesis through a series of epigenetic changes (176). CAFs have been extensively studied in the regulation of cancer generation and development. CAFs are a kind of heterogeneous cells with high plasticity, whose cellular characteristics and interactions with other cells vary with tumors and combine both tumor-promoting and tumor-suppressing functions (176). Inflammatory ligands, growth factors and extracellular matrix proteins secreted by CAFs in the tumor microenvironment further enhance the therapeutic resistance, proliferation and immune rejection of cancer cells, thereby promoting tumor progression and even causing malignant tumorigenesis (174).

Therefore, the analysis of specific cell surface markers of CAFs can provide deeper insight into the diversity of their cellular functions, while targeting therapies based on the characteristics of the markers is of great significance for both early cancer prevention and follow-up therapy. The cell surface markers of CAFs are usually applied for CAFs cell type identification, the alpha smooth muscle actin (αSMA) and fibroblast activation protein alpha (FAP) separate CAFs from other fibroblast cell pools, and more precise cell classification is what can improve the CAFs target identification accuracy (177). However, some scholars argue that CAFs markers have been restricted to only two extremely heterogeneous markers, αSMA and FAP, in past studies lacking some persuasive force. In further studies, CAFs cell populations have been discovered that they can be separated using PDGFRα/β and Thy-1 cell surface antigen (CD90), with GPR77 and CD10 as unique CAFs cell markers being able to maintain cancer cell stemness when promoting chemoresistance (178). In addition, CAFs heterogeneity is also related to multiple signal pathways. CAFs cells crosstalk with cancer cells by activating B-cell nuclear factor kappa-light-chain-enhancer signal pathways and other complicated signal networks, thereby reflecting their individual function characteristics (179). Targeting CAF cells functional signal pathways is also a means to kill cancer cells and heal cancer.

The natural products not only inhibit the proliferation of cancer cells, but also target stromal cells in the tumor microenvironment such as CAFs through various signaling pathways, thus suppressing proliferation and migration of desmoplastic tumors (14). A novel CAF population that expresses MHC class II and CD74 was discovered in pancreatic ductal adenocarcinoma, and this group of CAF cells can present antigen to CD4^+^ T cells (180). Many natural products possess immunoregulatory functions in the tumor microenvironment, and if the natural products can modulate the interaction between CAF cell groups and CD4^+^ T cells, they can regulate the pancreatic tumor immune response to kill cancer cells. So far, natural products inhibitory effects on CAF cells are mainly revealed in TGF-β signal pathway, but studies of WNT and Hh pathways are also important to inhibit desmoplastic tumor infiltration (14). Studies have found that low concentrations of curcumin could reverse tumor associated fibroblast activation to restore their normal state as well as inhibit *in vitro* pancreatic cancer cell migration and pancreatic cancer cell metastasis in the lung (181). Curcumin reduces the mesenchymal characteristics of CAFs to suppress pancreatic cancer migration and invasion, inducing phenotype reversal of epithelial-to-mesenchymal transition (EMT) in pancreatic cancer cells (182). In addition, it also finds that curcumin could not only reverses CAF cells phenotype into PTF cells, but also effectively reduces CAFs-mediated oncogenic actions of Cal27 by inhibiting TSCC CAFs (183). Resveratrol eliminates IL-6 secreted by CAFs to prevent Cholangiocarcinoma cells from migrating. Moreover, resveratrol induces cancer cell autophagy to reverse the cellular malignant transformation (184). Resveratrol in prostate cancer affects epithelial-stromal crosstalk in the tumor microenvironment by activating CAFs-associated TRPA1 ion channels, which ultimately promotes cancer cell apoptosis (185). Therefore, natural products can regulate TAMs by multiple signal pathways, using signal regulatory networks and specific cell surface targets as therapeutic routes may be an effective means to eliminate cancer cells.

### 3.6 Adipocyte

Cancer-associated adipocytes (CAAs) are closely linked to cancer occurrence and development. CAAs produces a local or larger scale effect by releasing various factors that further facilitate the development of tumor (186). Adipose tissue is able to secrete multiple hormones and chemokines that regulate the inflammatory response and the tumor microenvironment, while lipid homeostasis disorders caused by dysregulated lipid metabolism will further exacerbate cancer cell proliferation and metastasis (187). CAAs within or around tumor tissue as well as in distal tissues have been known to affect tumor development, migration, and resistance through endocrine, paracrine, and metabolic reprogramming (188). In addition, studies show that proinflammatory factors and macrophage counts are positively correlated with adipocyte size and that a vicious cycle between macrophages and adipocytes involving tumor necrosis factor-α and free fatty acids lead to an increased inflammatory response (189). The secretion of angiogenic factors, inflammatory factors and estrogen in adipocytes metabolism is strongly associated with breast cancer, while dyslipidemia, redox stress and insulin resistance caused by abnormal adipocytes metabolism may contribute to further cancer deterioration (190). The expression and secretion of adipocyte inflammatory molecules are altered in breast cancer with increased inflammatory factors TNF-α and IL-6 as well as chemokines CCL2 and CCL5, which directly results in hemangiogenesis in the tumor microenvironment along with cancer cell proliferation and metastasis (186). The breast cancer cell-macrophage-adipocyte relationship is interconnected *via* multiple reactive processes, notably: release of inflammatory factors, TSC1-TSC2 complex crosstalk with mTOR, insulin resistance, endoplasmic reticulum stress, oxidative stress, and elevated estrogen levels (189). The differentiation of bone marrow adipocytes is influenced by many factors, and they secrete various bioactive materials which can be used by cancer cells to trigger adenocarcinoma, breast cancer, lung cancer and multiple myeloma (191). Due to low success rates of modulating CAAs alone for cancer treatment, combination therapy using lipid metabolism inhibitors, cytostatic agents and targeted therapeutics has more potential to eliminate malignancies (188).

Natural products always have an outstanding regulatory function in the tumor microenvironment, so researchers conducted extensive studies on the effects of natural products on adipocytes cell cycle and regulatory factors. For example, a natural product of small molecules called Rutaecarpine is able to reduce the incidence of adipocyte-associated obesity by regulating the AMPK signal pathway (192). Cancer is a disease that affects the homeostasis of the whole organism. Diabetes and obesity caused by disturbed metabolism also present a great health risk for cancer patients. Treatment of human mesenchymal cells with oryzativols A dramatically enhances the expression of Runx2 and the differentiation of cells into osteoblasts, and it also suppresses gene expression of peroxisome proliferator activated receptor γ in mesenchymal stem cells (193). AMPK is a critical regulator of COX-2, ERK1/2 and p38 in cancer cells, curcumin controls the adipocytes and cancer cell survival by downregulating peroxisome proliferator-activated receptor-γ in adipocytes and COX-2 in MCF-7 cells upon stimulating AMPK (194). Curcumin inhibits the pro-inflammatory transcription factors nuclear factor kappa B and Wnt/beta-catenin and activates the Nrf2 cell signaling pathways and peroxisome proliferator-activated receptor-gamma, thereby downregulating tumor necrosis factor and monocyte chemotactic protein-1, as well as upregulating adiponectin and other gene products (195). Resveratrol inhibits adipogenesis by downregulating the fatty acid synthase (FAS) gene and upregulating the expression of pro-apoptotic genes BNIP3 and DAPK2, thus inducing cancer stem cell apoptosis, without showing obvious toxicity (196). Most natural products possess immunomodulatory functions, and modulating cancer immune system by CAAs is a mild way for natural products to treat cancer.

### 3.7 Inflammatory cytokines

Inflammatory cytokines are common cytokines in the tumor microenvironment that have a proven ability to regulate the inflammatory response in controlling tumor development, and thus have been explored as potential targets for cancer therapy. Inflammatory cytokines, as soluble proteins that mediates the information communication between cells, are capable of exerting immunomodulatory functions *via* signal transfer between cancer cells. Multiple types of cells, signal pathways, enzymes and cytokines comprise the complex inflammatory microenvironment of tumors, and inflammatory cytokines are critical targets for regulating the inflammatory microenvironment to treat cancer (197). Clinical trials have shown that the proinflammatory cytokines recombinant interferon-alpha and interleukin-2 have potent antitumor activity and could be used in various malignant tumor treatments (198). Researches reveal that under proinflammatory cytokines and chemokines, tumor necrosis factors and IL1b can activate CXCR4 receptors on prostate cancer cells to facilitate the invasion and metastasis of cancer cells (199). Normally, the quantity of inflammatory cytokines could be dramatically increased after blocking the programmed death (PD)-1/PD-L1 axis and activating immune cells such as natural killer cells, macrophages and T cells (200). In addition, a number of inflammatory cytokines including 12-o-tetradecyl foppo-13-acetate, ceramide, hydrogen peroxide, lipopolysaccharide and okadaic acid have been found able to induce cytokines TNFα and IL-1β production by lymphoma cells (U-937), fibroblasts (HFL1) and lung epithelial cells (A-549) (201). Inflammatory cytokines are intimately associated with multiple critical immune cells in the tumor microenvironment and choosing appropriate inflammatory cytokines as the targets of immunotherapy may be able to kill cancer cells more precisely. T cells are gradually depleted and exhausted in tumor tissues but targeting factors of inflammatory cytokine signal can enhance anti-tumor immune response activity by improving CAR-T cell and TCR transgenic T cell viability (202). In addition, a number of inflammatory cytokines, including 12-o-tetradecyl foppo-13-acetate, ceramide, hydrogen peroxide, lipopolysaccharide and okadaic acid, have been found to be able to induce cytokines TNFα and IL-1β production by lymphoma cells (U-937), fibroblasts (HFL1) and lung epithelial cells (A-549). The strategy of inflammatory cytokine targeting is failed to achieve satisfactory cancer therapeutic outcome, whereas the ability to target proteins or their encoding genes into the tumor microenvironment or cancer cells through a combination of cellular therapies, gene therapies, monoclonal antibodies and other drugs would achieve desirable cancer therapeutic effects (198).

Natural products widely used in cancer immunotherapy are also important in the regulation of inflammatory cytokines. Andrographolide directly inhibits NF-κB binding to promoter DNA and facilitates the secretion of inflammatory cytokines by regulating the gene expression (203). Natural products are safer than other radiotherapeutic and chemotherapeutic agents as well as being generally less resistant and toxic. Moreover, natural products usually exert antiangiogenic, antimetastatic and ant-inflammatory effects in the tumor microenvironment, thus making them significant candidates for cancer immunotherapy development. Resveratrol nanoparticles can inhibit the growth, proliferation and migration of cancer stem like cells by inducing M1-like macrophages producing inflammatory cytokines such as TNF-α, IL-6 and IL-1β (204). Pluronic block copolymer encapsulated curcumin has been demonstrated to improve the stability, solubility and sustained release of curcumin, and Pluronic micelles encapsulated curcumin promoted apoptosis by decreasing inflammatory cytokine release and inhibiting NF-κB signal pathway activation to block cell cycle in G0/G1 phase (205). Human bronchial epithelial cells treated with berberine are unaffected by MAP kinase pathway and NF-κB pathway, but nuclear STAT6 protein expression is dramatically decreased since berberine inhibited the inflammatory cytokines CCL11 and IL-6 thereby regulating STAT6 signal pathway (206). Berberine suppresses cytokines induced by inflammatory cytokines *via* regulating the phosphorylation and degradation of inhibitory kappaBα (201). Activation of cytokines and NF-κB signal pathways facilitate cancer cell deterioration and metastasis (207). However, natural products have incalculable potential to regulate immune responses in the tumor microenvironment by inflammatory cytokines. Therefore, finding appropriate specific targets, clarifying inflammatory cytokines regulation routes and adopting novel techniques to realize highly efficient conversion of the natural products are practical approaches for many types of cancer immunotherapy.

## 4 Natural products regulate the tumor microenvironment to overcome resistance

### 4.1 Tumor treatment resistance

With the improvement of cancer treatment technology, new treatment agents are emerging. Radiotherapy and chemotherapy agents have shown a severe resistance problem though could eliminate a huge amount of cancer cells. Declining survival rates for cancer patients due to tumor progression and cancer recurrence which arising from cancer treatment resistance is a challenge that plagues the medical community. Patients with metastatic breast cancer often have resistance to chemotherapy as well as biologic therapy, and many advanced breast cancer patients suffer from multidrug resistance due to overexpression of drug transporters or βIII-tubulin soforms, resulting in a critical requirement for novel agents effective in tumor resistance (208). While non-small cell lung cancer patients undergo empiric chemotherapy after surgical resection, lung cancer have varying degrees resistant to doublets–carboplatin and paclitaxel, cisplatin and gemcitabine, cisplatin and docetaxel, cisplatin and navelbline chemotherapeutic agents (209). Chemotherapy resistance mechanisms in gastric cancer are complicated, mainly due to the interaction between cancer cells and the tumor microenvironment, dysregulation of cell survival and death signaling pathways, altered drug targets, and reduced drug concentrations (210). Tumor resistance to androgen receptor (AR) targeted chemotherapy in metastatic castration resistant prostate cancer (MCPRC) due to intratumoral androgen mutation, expansion, steroid increase, AR splice variant expression, and androgen independent tumor cell development (211). Primary resistance or acquired resistance from chemotherapy is a major reason for clinical anti-cancer treatment failure and finding the anti-cancer products with different treatment mechanisms from chemotherapy agents is the main research direction for cancer treatment.

### 4.2 The tumor microenvironment and resistance

In the complicated tumor microenvironment, tumor cells, tumor stroma and immune cells interact and communicate with each other which gradually builds up resistance of cancer cells. The tumor microenvironment is commonly associated with cancer therapy-acquired resistance generation due to CAFs, highlights myeloid cells, and mesenchymal stem cells that are able to form cancer cell resistance signal pathways in the tumor microenvironment (212). Resistance is induced by tumor or stromal cells which secrete soluble factors in the tumor microenvironment, and the activation status of immune cells in the tumor microenvironment also affects the resistance generation (213). Extracellular vesicles represent an important mechanism for exchange bioactive molecules among cells, and changes in nucleic acid cargoes, proteins and lipids caused by the crosstalk of the tumor microenvironment and extracellular vesicles eventually lead cancer cells to acquire drug resistance (214, 215). Cisplatin is a commonly agent utilized in many types of cancer treatment, while the tumor microenvironment has a significant effect on the generation of cisplatin resistance. A study finds that cisplatin resistance is significantly reduced when being combined with novel agents that target components of the tumor microenvironment such as immune cells and angiogenic factors (216). The success of this regimen in clinical treatment confirms the feasibility of modulating tumor microenvironment to assist reducing cisplatin resistance. Melanoma cells exhibit resistance in both chemotherapy and targeted therapy, while tumor cells and tumor stroma binding can form an inflammatory tumor microenvironment, targeting the tumor microenvironment with multimodal therapies has potential to attenuate drug resistance (217). Therefore, searching for appropriate agents to target the regulation of bioactive molecules and immune responses in the tumor microenvironment may solve the issue of previous agents that generate resistance in cancer treatment.

### 4.3 Natural products overcoming resistance

Natural products have been used in the treatment of cancer because of their versatility and lower side effects. Natural products generally have more than one action target which play a significant role in reducing radiotherapy-chemotherapy side effects, improving immunity and inhibiting tumor proliferation (218). Multiple studies indicate that natural products may have an important contribution to address multidrug resistance issues that arise in cancer pharmacotherapy. Natural products are able to act on receptors, enzymes, signal pathways and other biological targets, which in turn regulate tumor cell metabolism, cell migration, oxidative stress, inflammatory response and angiogenesis (219). Multidrug resistance can be caused by a variety of factors, including changes in drug metabolic enzymes, target proteins, and ATP-binding cassette transporters, or changes in apoptotic signaling pathways in tumor cells, which may reduce cancer treatment success (218). Different types of resistance markers have variable sensitivity to natural products. For example, topoisomerases and protein kinase C are relatively insensitive to natural products, while breast cancer resistance proteins and P-glycoproteins are frequently used as action targets of natural products for cancer treatment (220). Breast cancer resistance protein (BCRP/ABCG2), multidrug resistance-associated protein 1 (MRP1/ABCC1) and P-glycoprotein are major substances to induce multidrug resistance, while alkaloids, coumarins, flavonoids and terpenoids may be the natural products that can solve multidrug resistance problems (221). Studies find that lung cancer treatment with natural products such as resveratrol, berberine, ginsenosides and silymarin can significantly reduce drug resistance in comparison to conventional treatment with regorafenib, sorafenib and ramoximab (219).

The main routes of resistance in tumors include the Renin-angiotensin system (Ras), Phosphatidylinositol-3-kinase/protein kinase B (PI3K/Akt), Epidermal growth factor receptor (EGFR), Transforming growth factor-beta (TGF-β), Notch, and Wnt signal pathways (222). Exploiting natural products that have an inhibitory effect on carcinogenic or cancer-promoting signal pathways is one of strategies to prevent drug resistance in cancer treatment. Natural products can not only modulate various drug resistance signal pathways in breast cancer stem cells, but they also have potential in targeted modification of breast cancer stem cell structures (223). If a natural product becomes available new agents to target breast cancer stem cells, it will greatly diminish the risk associated with resistance in breast cancer treatment. Studies have found that multidrug resistance is mainly caused by the premature efflux of drugs from cancer cells mediated by the ATP-binding cassette (ABC) transporter P-glycoprotein (224). While conventional targeted therapeutics struggle to modulate the complex multidrug resistance signal pathways in cancer cells, natural products have the ability to target and modulate multidrug resistance mechanisms (225). Various researches have proven that natural products such as pomegranate extracts and citronellol have anti-tumor functions and many marine derived compounds which contain poloxysterols, alkaloids, peptides and terpenoids can reverse multidrug resistance (224, 226). In addition, combination therapy with natural products and chemotherapeutic agents effectively enhances the sensitivity of tumor cells to chemotherapeutics, using nanocarriers to deliver natural products and chemotherapeutic agents maximizes synergistic effects against multidrug resistance (225). Leveraging natural products to target and modulate multidrug resistance signal pathways will improve the cancer therapeutics efficacy and reduce deaths in cancer patients.

### 4.4 Natural products regulate the tumor microenvironment to overcome resistance

The tumor microenvironment contains multiple types of cells which secrete cytokines to mediate signaling pathways and affect the cancer proliferation. As the space that cancer cells depend on for survival, the changes of tumor microenvironment also affect the emergence and development of drug resistance. As known, the tumor microenvironment-mediated drug resistance arising often relates to cancer cells and cellular stromal composition (227). The tumor microenvironment induces drug resistance mainly through cytokines secreted by stromal cells or tumor cells, and adhesion molecules secreted by extracellular matrix and fibroblasts also diminish the drug therapeutic effect (228). Resistance that occurs in cancer therapy is intimately linked to the tumor microenvironment, and such changes, which are not genetic and epigenetic inheritance, are most likely a non-cell-autonomous mechanism of resistance (227). The tumor microenvironment has a high level of interleukin-6 (IL-6), which is a growth factor to stimulates cell growth and also generates some degrees of resistance in radiotherapy and chemotherapy (229). IL-6 cytokines that derive from the tumor microenvironment combine with the downstream IL-6/STAT3 signal pathway to constitute a central regulator in chemotherapeutic response (230). In research on human papillomavirus type 16 (HPV16) tumor models, IL-6 secreted by tumor cells was found separately to have resistance to cisplatin chemotherapy and HPV16 vaccine immunotherapy (231). Therefore, selecting appropriate agents to regulate the secretion of IL-6 cytokines and the signal pathways that they participate in may decrease the resistance. Research has found that IL-6 paracrine loop in breast cancer cells responds to chemotherapeutic drugs, but iron metabolism can break this IL-6 local niche in the tumor microenvironment thus blocking the IL-6 signal pathway to overcome chemoresistance (229). TAMs promote phagocytosis and oxidation drugs in the tumor microenvironment to fully exploit their anticancer functions and contribute to improving the tumor microenvironment metabolic levels against drug resistance (232).

Previous research has shown that natural products can target the regulation of cytokines and immune responses in the tumor microenvironment *via* multiple signaling pathways, which is an excellent option to address the tumor drug resistance problem. Natural products can regulate the immune response or reshape the tumor inflammatory microenvironment to combat the side effects of oncology drug resistance. A study on traditional Chinese medicine anti-tumor found that three typical natural products of Huangqin, oroxylin A, wogonin and baicalin, may overcome anti-cancer drug resistance by modulating the tumor immunosuppressive microenvironment (233). Natural products modulate immune checkpoint-related signaling molecules *via* multiple signaling cascades in the tumor microenvironment combined with immune checkpoint inhibitors (ICIs), which not only enhance the effectiveness of immunotherapy but also reduce the adverse effects of resistance (234). Signal transducer and activator of transcription 3 (STAT3) hyperactivation regulates immune responses in the tumor microenvironment by cytokines, growth factors, and G protein-coupled receptors to attenuate drug resistance and control cancer metastasis (235). However, natural products can target to oncogenic signaling molecules STAT3, which may affect STAT3-associated PD-1+CD8+ T cells and IL-6/STAT3/PD-1 transcription regulation systems in the tumor microenvironment, ultimately controlling the generation and progression of drug resistance (235, 236). All in all, natural products are closely related to the immune response and cytokines in the tumor microenvironment, regulating the tumor microenvironment state to control drug resistance by natural products might achieve safer and more stable clinical treatment effects in cancer.

## 5 Summary and prospect

Natural products participate in regulating multiple signal pathways in the tumor microenvironment to exert anti-tumor functions from the genetic, epigenetic, molecular, and cellular levels (11). Natural products are frequently favored by researchers for their low toxicity and multi-target characteristics in tumor therapies, thus more natural products are being investigated for anti-tumor functions. In our previous study, resveratrol was found to promote endoplasmic reticulum (ER) stress response, cellular autophagy and apoptosis in gastric cancer cells at a dose-dependent manner. Moreover, the combination of resveratrol and endoplasmic reticulum stress activator further stimulated cancer cell death (237). Melatonin, a hormone synthesized and secreted mainly by the pineal gland, could prevent cancer survival *via* modulating ER stress, autophagy, and RAS/RAF/ERK signaling (238). Furthermore, enhancement of resveratrol-inhibited cancer cell proliferation and migration could be achieved by regulating lncRNA expression and ER stress (237). Actually, natural products such as resveratrol have the capacity to modulate immune cells and immune factors, which further authenticates the conjecture that natural products can reshape the tumor microenvironment to stimulate the death of cancer cells. Alternatively, besides subjecting cancer cells to cell death, it would be possible that applying proper strategy, such as using natural products, guides cancer cells to death or differentiate malignant to benign cells (239). It is similar to the concept of “viewing the situation as a whole” in traditional Chinese medicine.

The efficacy of most clinical anti-cancer agents is interfered by drug resistance, which directly results in reduced cure rates and prolonged treatment duration for cancer patients. The mechanisms by which drug resistance arises are complex and involve multiple cell types, cytokines, as well as signal regulatory networks. The development of drug resistance in cancer cells cannot be separated from the regulation of the tumor microenvironment, in which the interaction of immune cells, tumor stroma and tumor cells gradually establishes resistance of cancer cells. In the study of overcoming drug resistance in cancer cells, we found that the combination of cisplatin and magnesium chloride (MgCl_2_) could regulate the Wnt/β-catenin signaling pathway to enhance the cisplatin effect and use exosomes to control ferroptosis was also effectively inhibited cancer (240, 241). Targeted drugs are often considered to be efficient therapies for cancer, but unfortunately, targeted therapies are all subject to varying degrees of resistance. Therefore, utilizing the natural products to regulate the tumor microenvironment with targeted drugs for maximum therapeutic effect is a plausible approach to change the drug resistance condition.

Natural products in anticancer applications have problems such as poor targeting and transport difficulties, along with complex action mechanisms and influencing factors. Consequently, further clarification of the action mechanisms and crucial regulatory factors for each type of natural product in the tumor microenvironment is desired. Besides the combination treatment of natural products with conventional drugs to improve the therapeutic efficacy, there are possibilities to achieve new breakthroughs by gene editing technology, nanotechnology and so on. The exosomal molecular delivery system may assist natural products in their anti-tumor function and using gene editing technology also have potential to enhance the therapeutic effect of natural products (11). Nanoformulations can improve the accuracy when natural products being transported and released, while nanoformulations with transcytosis capability can further promote the depth of tumor penetration (242). CRISPR/Cas9 editing technology, when combined with various systems biology tools and sequencing systems, is able to modify target genes to optimize gene expression systems (243). Knock-in or knock-out effector genes of natural products by CRISPR/Cas9 editing technology in the tumor microenvironment, along with the use of exosomal molecular delivery systems and nanoformulations to improve the transfer efficiency of natural products, which may release tremendous anti-cancer potential of natural products. Relevant experimental studies are still rare, but I believe that as researchers keep researching, the puzzle will be unraveled and explored more efficient anticancer methods of natural products.

## Author contributions

WZ and YH drafted the manuscript; SL and YH revised the draft; CL and TL made substantial contributions to the work through in-depth discussion. All the authors proposed the Research Topic theme, made a direct and intellectual contribution to the work, and approved the final version for publication.

## Funding

This study was funded by the National Natural Science Foundation of China (Nos.81502582). Funding was also provided by the Fundamental Research Funds for the Central Universities (N182004002), Natural Science Foundation of Liaoning Province (2021-MS-104, 2022-YGJC-39), Fundamental Scientific Research Fund of Liaoning Provincial Education Department (LJKQZ2021002), and Liaoning Revitalization Talents Program, Key technologies research in major disease control and prevention (XLYC2007098).

## Conflict of interest

The authors declare that the research was conducted in the absence of any commercial or financial relationships that could be construed as a potential conflict of interest.

## Publisher’s note

All claims expressed in this article are solely those of the authors and do not necessarily represent those of their affiliated organizations, or those of the publisher, the editors and the reviewers. Any product that may be evaluated in this article, or claim that may be made by its manufacturer, is not guaranteed or endorsed by the publisher.
